# Characterisation of 3D Bioprinted Human Breast Cancer Model for In Vitro Drug and Metabolic Targeting

**DOI:** 10.3390/ijms23137444

**Published:** 2022-07-04

**Authors:** Titanilla Dankó, Gábor Petővári, Regina Raffay, Dániel Sztankovics, Dorottya Moldvai, Enikő Vetlényi, Ildikó Krencz, András Rókusz, Krisztina Sipos, Tamás Visnovitz, Judit Pápay, Anna Sebestyén

**Affiliations:** 1 Department of Pathology and Experimental Cancer Research, Semmelweis University, Üllői út 26, 1085 Budapest, Hungary; tita.danko@gmail.com (T.D.); gaborpetovari@gmail.com (G.P.); regiraffay@gmail.com (R.R.); sztankovics.daniel@gmail.com (D.S.); moldvai.dorottya@gmail.com (D.M.); eniko.vetlenyi@gmail.com (E.V.); krencz.ildiko@gmail.com (I.K.); rokusz.andras@med.semmelweis-univ.hu (A.R.); krisztina.sipos.13@gmail.com (K.S.); papay.judit@med.semmelweis-univ.hu (J.P.); 2 Department of Genetics, Cell- and Immunobiology, Semmelweis University, Nagyvárad tér 4, 1089 Budapest, Hungary; tamas.visnovitz@gmail.com; 3 Department of Plant Physiology and Molecular Plant Biology, ELTE Eötvös Loránd University, Pázmány Péter Sétány 1/c, 1117 Budapest, Hungary

**Keywords:** 3D bioprinting, breast cancer models, tissue heterogeneity, xenograft, drug response

## Abstract

Monolayer cultures, the less standard three-dimensional (3D) culturing systems, and xenografts are the main tools used in current basic and drug development studies of cancer research. The aim of biofabrication is to design and construct a more representative in vivo 3D environment, replacing two-dimensional (2D) cell cultures. Here, we aim to provide a complex comparative analysis of 2D and 3D spheroid culturing, and 3D bioprinted and xenografted breast cancer models. We established a protocol to produce alginate-based hydrogel bioink for 3D bioprinting and the long-term culturing of tumour cells in vitro. Cell proliferation and tumourigenicity were assessed with various tests. Additionally, the results of rapamycin, doxycycline and doxorubicin monotreatments and combinations were also compared. The sensitivity and protein expression profile of 3D bioprinted tissue-mimetic scaffolds showed the highest similarity to the less drug-sensitive xenograft models. Several metabolic protein expressions were examined, and the in situ tissue heterogeneity representing the characteristics of human breast cancers was also verified in 3D bioprinted and cultured tissue-mimetic structures. Our results provide additional steps in the direction of representing in vivo 3D situations in in vitro studies. Future use of these models could help to reduce the number of animal experiments and increase the success rate of clinical phase trials.

## 1. Introduction

Tissue cell culturing and tumour cell lines as model systems are very important in basic research and are necessary for drug discovery and therapeutic developments. Cell culturing and tumour cell line establishment have a long history, spanning more than a 100 years. Hanging drop (HD) technology started to be used in microbiology as one of the first developed methods for traditional 3D culturing, and it was adapted to neural tissue cultures by *Harrison*, then was used further in neurobiology and embryology [1,2]. *Levi-Montalcini* observed guided ganglia development in the presence of nerve growth-inducing tumours using HDs in chick embryos; this discovery of growth factors was awarded the 1986 Nobel Prize for Physiology or Medicine (shared with *Cohen*) [3]. The HD technique has had a renaissance; it is a widely used tissue culturing method for studying stem cells, tumour cells without flattening to the surface of culturing plates, and three-dimensional (3D) structures in the development of tissues and diseases [1]. Human tumour cells began to be established in the middle of the last century, beginning with HeLa in two-dimensional (2D) cell cultures. Despite the possibilities of developing new 3D culturing procedures, and the many limitations of 2D cell lines, the traditional monolayer cultures are still predominant in cellular assays and high-throughput screening technology, being used in both basic and translational cancer drug developments [4,5,6]. Most drug compounds fail due to a lack of efficacy or safety in clinical phase studies, especially in cancer research. The low success rate and the high costs of clinical trials have proved the urgent need for new preclinical models, which better represent the in vivo environmental situation. From the 1980s, many research teams have highlighted the importance of extracellular matrix (ECM) in tumour tissues; it is well-accepted that in 3D structures and the tissue microenvironment, the complexity needs to be considered in the development of therapy resistance, which is the main problem in cancer treatments [7,8].

Cellular, genetic, and microenvironmental diversity and heterogeneity contribute to tumour development and therapy resistance. Providing the requirements of the bioenergetic background of tumour growth (energy, nutrients, and building blocks), metabolic heterogeneity, symbiosis and flexibility/plasticity-mediated adaptation mechanisms in cancer tissue progression have also been highlighted in cancer research [9]. The “Warburg effect”—high-grade glucose uptake with significant lactate production—was named after *Otto Warburg* in the 1970s [10]. *Warburg’s* results and views were reconsidered in recent decades, and in addition, characteristic hallmarks of cancer were revealed with deregulated cellular metabolism and the unlocking of phenotypic plasticity by *Hanahan* and *Weinberg* [11]. The Warburg effect/Warburg phenotype has characteristic expression and activity changes in many metabolic enzymes and transporters. As a consequence, the increased amount of glycolytic metabolites can fuel the pentose phosphate and other macromolecule synthetic pathways for fast-growing cells (nucleotide, protein and fatty synthesis). Moreover, lactate is not only a by-product, but it is an important oncometabolite, even increasing microenvironmental acidification, which has further impacts on tumour progression, therapy response and metastasis (e.g., on matrix remodelling and the immune microenvironment). Lactate contributes to metabolic symbiosis, and can be used and oxidized by highly oxygenated tumour cells or fibroblasts with higher oxidative phosphorylation (OXPHOS). activity. Recently, attention has also been paid to the provision of fatty acid, acetate, citrate, and amino acid utilization processes in these cells. Additionally, besides glucose, amino acids could be important carbon sources for proliferating cells in tumour tissues. For example, glutamine utilization and anaplerosis through the TCA cycle can support the growth and/or survival of tumour cells under hypoxia. Based on these findings, besides cancer cells with a glycolytic/Warburg phenotype (using the Warburg effect with a truncated TCA cycle and glutaminolysis), tumour cells with other metabolic phenotypes can be distinguished, especially in cancer tissues, including OXPHOS metabolic characteristics (another main phenotype with higher mitochondrial OXPHOS and increased mitochondrial content and enzyme expression) and the most dangerous hybrid state (with the high flexibility to utilise both OXPHOS and Warburg glycolysis simultaneously). However, these main phenotypes could contribute to the continuous transitions of the metabolic features of tumour and non-tumour (immune cells, fibroblast, adipocytes, etc.) cells forming tumour tissue (phenotypes and the studied proteins are shown in Supplementary Figure S1).

Many cancer hallmarks (e.g., deregulated metabolism, phenotypic plasticity, and tumour-promoting inflammation), including metabolic adaption mechanisms and tissue heterogeneity, cannot be represented in traditional cell cultures [12]. Local and time-dependent heterogeneity, tumour tissue symbiosis, plasticity (which is a newly identified hallmark), and tumour evolution cannot be created in traditional in vitro models, where we tried to achieve “simple, well-organised and controlled monoclonal, monomorph” tumour-cell-derived cell cultures [13]. Recent drug targets were mainly identified and validated from basic research using 2D tumour cell cultures or xenograft experiments with no real structure, no tissue formation or without real human ECM [5]. Drug resistance has a special importance in recent therapy developments focusing on cellular heterogeneity, interconnections between cells, several limiting molecular concentration gradients, ECM composition and matrix stiffness, which could influence the drug response in the tissue microenvironment [6]. Dynamic changes in ECM molecules regulate many cellular functions, including differentiation, migration, adhesion, proliferation, and survival. These could contribute to several diseases (e.g., fibrosis and cancer) and therapeutic failures (such as cancer drug resistance) [4,14,15]. However, biochemical composition is very important, and the physical and mechanical characteristics (e.g., rigidity) of ECM are also important in maintaining tumour development [16,17,18]. The comparison of the recently developed 3D culturing technology and with 2D cell cultures and their drug sensitivity test results has shown that 3D models can more reliably show the in vivo characteristics of cancer cells. The reduced drug sensitivity of 3D cell cultures (mainly the same in vivo) [19] with reduced proliferation [20], altered gene expressions [21], morphology [22], and invasion [23] were described in many study models and tumour cell types dependently. Based on these findings, these systems can be suggested for the promotion of pharmaceutical developments and the testing of new drug candidates. These traditional 3D culturing systems (including HD, matrix-embedded 3D, ultra-low attachment (ULA) plates, and organoid cultures) can represent cell–cell interactions, some cell morphology changes, and ECM synthesis, composition, and drug sensitivity better than 2D models; however, these systems have diverse and model-type-dependent in vivo imitation, pure reproducibility, and high cost (especially the organoids) [19]. The use of 3D bioprinted models is emerging, suggesting that test results could be standardised and more reproducible in the future.

Tumours, especially breast cancers (BCs), have many distinct subtypes. As an example, the most common invasive ductal carcinoma begins with an overgrowth of ductal epithelial cells. Finally, tumour cells invade into neighbouring tissues, blood, and lymph vessels, migrate, and form tumours in new places depending on their aggressiveness. It is also well-known and described that tissue heterogeneity contributes to these processes during progression. To model these processes in monolayer cultures is almost impossible, as only certain steps can be investigated using 2D cell cultures [24,25]. Three-dimensional bioprinting is a promising technology using ECM-compatible biomaterials and cells to create complex in vitro tissue-mimetic models, allowing longer culture. The development of 3D-printed models and in vitro culturing will help build more complex models with more reproducibility than organoid and spheroid cell cultures [26,27,28]. In 3D bioprinting, the 3D structure can be precisely guided and cells and materials can be selected and altered using different, defined matrixes and cell types [29]. There are several studies that proved that invasive BCs deposit and restructure their stromal ECM with collagen [30], e.g., collagen spheroid invasion studies underlined the role of a multistage program where individual leader cells initiate the invasion and the follower cells expand in the 3D microenvironment with fibrillary collagen-I-rich environments [31]. Therefore, several new 3D breast cancer models have started to be used widely in different experiments [32,33]. These models highlight changes in cell behaviour between cancer cell growth in 2D and 3D models, including altered proliferation, gene expression, and chemo- and radiosensitivity [34,35]. Therefore, the goal of 3D models is to retain cellular behaviour as closely as possible to that of in vivo tissues and simultaneously preserve investigational possibilities, studying signalling alterations and tumour development with regard to drug responsiveness in particular. Comparing traditional 2D drug sensitivities to 3D models usually results in differences; however, a complete study comparing 2D, 3D, and in vivo xenografts, using the same model cell lines, drug, and protein expression analyses and the in situ tissue heterogeneity of some markers, is missing [34].

In the presented study, we tested a 3D bioprinted in vitro model of the luminal BC cell line (ZR75.1). Using the previously selected alginate-based bioinks, which were compatible for bioprinting several BC cells [36,37], we constructed ZR75.1 tissue-mimetic scaffolds (TMSs) combining scaffolds and cell-containing bioink layers, and incubated these structures for a minimum of 3 days. In these conditions, the growing and survival capacities of the printed TMSs were tested by proliferation and viability tests at different time points. The tumorigenic capacity and morphology of the evolving tissue-mimetic structures were tested using subcutaneous xenotransplantation in SCID mice and formalin-fixed, paraffin-embedded tissues for pathomorphology and confocal microscopy examination. We compared the in vitro drug sensitivity and characterised the mTOR complex/metabolic enzyme expressions of 2D cell culture systems, 3D bioprinted in vitro TMSs, and other 3D spheroid cultures in the same cell-line-derived in vivo xenografts. Based on our results, the established 3D bioprinted in vitro model system is suitable for future drug sensitivity and tumour metabolism studies. In these aspects, we first described this in vitro 3D printable model, and confirmed that it shows comparable drug sensitivity and a similar mTOR/metabolic protein expression profile to the in vivo situation, compared with previously established 3D cell culture systems and traditional 2D cell cultures. Additionally, regarding our immunostainings, this suggested 3D bioprinted model can also represent the heterogeneity of in vivo tumours.

## 2. Results

### 2.1. Tissue Heterogeneity of Metabolic Enzyme Expression

**Figure 1:**
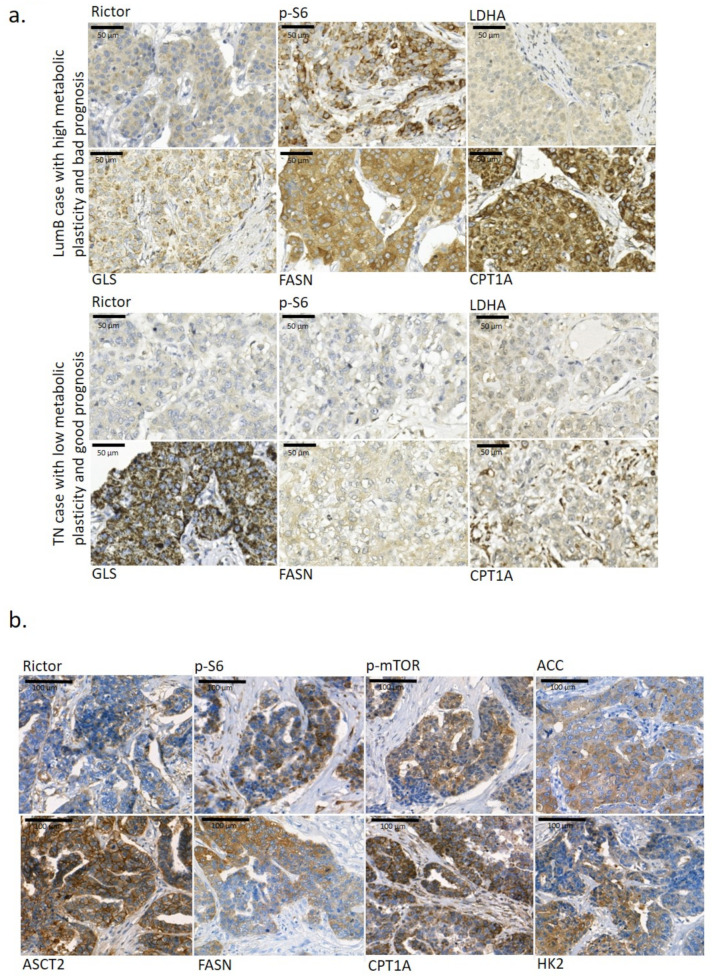
Metabolic enzyme expression heterogeneity in the studied breast cancer tissues. (**a**) Metabolic plasticity in tumour tissue and its importance in the overall survival of breast cancer patients. Two breast cancer cases with different metabolic plasticity at the tissue level—the Luminal B (LumB) case with high metabolic plasticity (based on Rictor, p-S6, GLS, FASN and CPT1A IHC staining and their high staining intensity) had a short overall survival of only 14 months; however, in another triple-negative breast cancer (TN) case with low metabolic plasticity, a much longer overall survival period was observed (201 months). High metabolic plasticity was considered if high mTOR activity was accompanied with intensive staining of a minimum of two studied metabolism-related enzymes. (**b**) Representative staining of several metabolic enzymes (Rictor, p-S6, p-mTOR, ACC—acetyl-CoA carboxylase, ASCT2—alanine, serine, cysteine-preferring transporter 2, FASN—fatty acid synthase, CPT1A—carnitine palmitoyltransferase 1A, and HK2—hexokinase 2) showed high tissue heterogeneity in the studied breast cancer cases. 3,3′-diaminobenzidine (DAB) (brown) was used as a chromogen. Scale bars are given in the figures.

Pathologists face tissue heterogeneity during diagnostic evaluation. The metabolic characteristics and metabolic tissue heterogeneity of cancer tissues can be studied with several methods (e.g., immunohistochemistry—IHC). We scored metabolic plasticity based on the metabolic enzyme expression (six metabolic protein markers) of human breast cancers with previously applied metabolic protein immunostainings (p-S6, Rictor, LDHA—lactate dehydrogenase A, GLS—glutaminase, CPT1A—carnitine palmitoyltransferase 1A, and FASN—fatty acid synthase) [38]. Regarding our described observations, we detected high/low metabolic plasticity in correlation with worse/good prognosis. Additionally, we observed intra-tumoural heterogeneity in cellular staining intensities independent of breast cancer type (Figure 1). In our current work, we studied the expression pattern heterogeneity of mTOR activity (p-mTOR, p-S6, Rictor), glycolysis (GLUT1—glucose transporter 1, LDHA, HK2—hexokinase 2, and PFKP—phosphofructokinase), glutaminolysis (ASCT2—alanine, serine, cytein-preferring transporter 2, and GLS), and other metabolic pathway markers (ATPb—β-F1-ATPase, CPT1A, FASN, ACSS2—acyl-CoA synthetase short-chain family member 2, and ACC—acetyl CoA carboxylase) by IHC in a representative BC tissue panel (n = 40, 10 from each main breast cancer subtype).

Some of these and other metabolic marker expressions were also evaluated using the available stainings in the Human Protein Atlas database (n = 7–12 depending on the antibody staining). Heterogeneous in situ protein expression was defined if intra-tumoural heterogeneity was detected in more than 40% of the evaluated tissue samples in the cases of certain IHC staining. In these analyses, we observed high intra-tumoral heterogeneity in 10 different metabolic marker stainings (Table 1). Only ATPb, LDHA, GLS and ACSS2 showed lower tissue heterogeneity in the majority of the studied IHC cases. However, most of the BC cases found in Human Protein Atlas database are classified as HER2+, and our evaluation confirmed almost all previously heterogeneous IHC staining results. Only ATPb and CPT1A staining patterns showed other features in cases from the Human Protein Atlas (Table 1). Additionally, the database evaluation called for our attention on further metabolic differences (increased heterogeneity) at the tissue level (e.g., PKM—pyruvate kinase isoenzyme M2—CAB019421, MCT1—monocarboxylate transporter 1—HPA003324, PDHB—pyruvate dehydrogenase E1 subunit beta—HPA036745, SDHA—succinate dehydrogenase complex flavoprotein subunit A—CAB034929).

### 2.2. Sensitivity and Metabolic Enzyme Expression Differences in 2D and 3D Spheroid Cultured ZR75.1 Cells and In Vivo Models

**Figure 2:**
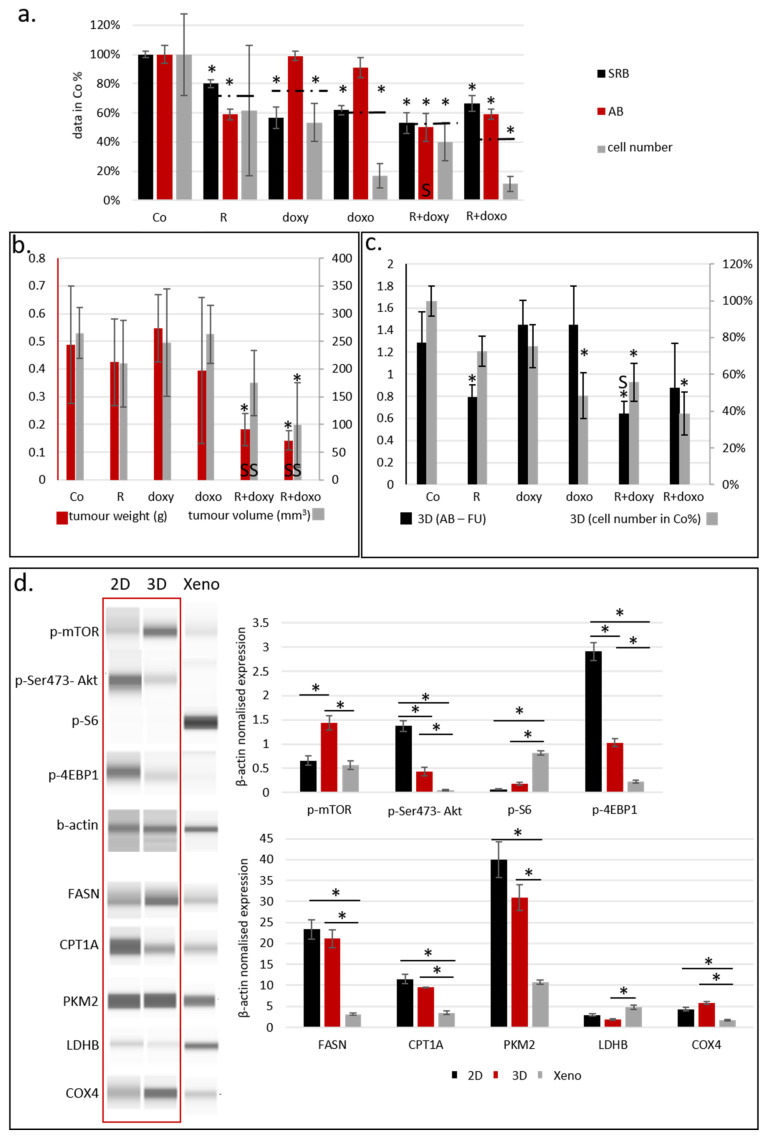
Sensitivity and metabolic enzyme activity differences of monolayer cultures, 3D-cultured spheroids (cells growing in hanging drops or ultra-low attachment plates) in vitro and the in vivo xenograft model. (**a**) In vitro proliferation results (sulforhodamine B (SRB) and Alamar blue (AB) tests, and cell counting) of ZR75.1 monolayer cell culture after 72-h treatments. Dashed lines show average viability % calculated from the three tests used. All data represent mean ± SD. Results are given in control %. (**b**) The registered tumour weights and the calculated final tumour volumes of the ZR75.1 xenografts after 21-day treatments. (**c**) Alamar blue (AB) assay and cell counting were used to detect the in vitro cell growth of ZR75.1 spheroids cultured in ULA plates (3D) after 72 h treatments. All data represent mean ± SD. Results are given in control %. (Co—control; R—rapamycin, 50 ng/mL or Rapamune 3 mg/kg; doxy—doxycycline, 10 µM or 5 mg/kg; doxo—doxorubicin, 50 ng/mL or 2 mg/kg). All data represent mean ± SD. Synergistic treatment interaction is labelled with S (based on combination index calculation). * *p* < 0.05. (**d**) Maintaining condition (monolayer—2D; HD spheroid—3D; xenograft—Xeno) affects protein expression pattern of ZR75.1 cells and xenograft tumour. Wes^TM^ Simple technique was used to detect mTOR activity (p-mTOR, p-(Ser473)-Akt, p-S6, p-4EBP1) and metabolic enzyme (FASN—fatty acid synthase, CPT1A—carnitine palmitoyltransferase 1A, PKM2—pyruvate kinase M2, LDHB—lactate dehydrogenase B, COX4—cytochrome c oxidase subunit 4) expressions (left panel). Densitometric analysis was performed to present the normalized protein expression differences (right panel) (β-actin was used as loading control). Red frame highlights the similarities of the studied enzyme expressions of 2D and spheroid cultures and underlines the differences in protein expression pattern obtained using ZR75.1 in vivo model. * *p* < 0.05.

The above-described metabolic heterogeneity of tissues is hardly represented in traditional 2D cell cultures, where the majority of proteins have homogenous expression, except for some (e.g., cell-cycle-dependent proteins such as Ki-67, cyclines, or, as we described previously, p-S6, which showed significantly higher expression in mitotic cells in a cell-type-dependent manner) [39]. Therefore, 3D cell culturing has begun to be applied broadly in many research works. In our previous study, we compared the metabolic enzyme expression of 2D BC cell lines grown in vitro and mTOR, GLS, glycolysis, and lipid metabolism inhibitor sensitivity. These results highlight that metabolic inhibitor combinations—e.g., rapamycin and doxycycline treatments—could have significant anti-proliferative effects in in vitro 2D cultured BC cell lines [40]. Therapy resistance could occur and, as a consequence, the drug sensitivity of cancer cell lines could be decreased with a higher passage number in in vitro cultured cells or in xenografts in vivo [41]. In our studies, we detected the lowered doxorubicin sensitivity of ZR75.1 in vivo. In this work, we compared the rapamycin (it has mainly anti-metabolic and drug-sensitizing effects), doxycycline (antibiotics targeting mitochondria) and doxorubicin (chemotherapeutics) sensitivity of in vitro 2D cultures and ZR75.1 breast cancer cells grown in vivo, and tried to increase the sensitivity using treatment combinations. After a 72 h treatment, two different proliferation tests (Alamar blue—AB and sulforhodamine B—SRB) were applied, and we controlled the detected differences by cell counting, as well. Finally, we registered rapamycin, doxycycline and doxorubicin sensitivity in vitro in ZR75.1 cells, and, as expected, the combinations were the most effective treatments (Figure 2a). In parallel, the xenotransplanted ZR75.1 cells were less sensitive for mono-treatments in a 21-day period. The registered tumour volumes and the final tumour weights showed that these cells could be resistant in vivo; however, rapamycin combination liberated the sensitivity in our in vivo experiments (Figure 2b). To test the role of 2D and 3D cell culture conditions regarding drug sensitivity and metabolic differences, traditional 3D cell cultures (ULA plates and HD cultures) were also tested. To identify the drug-treatment-induced effect in these conditions, we applied the AB test and cell counting in ULA plates. In HD cultures, only cell counting was applicable to detect the growth effects of treatments, and the obtained results were highly variable (data not shown). Greater differences were detected in the case of doxorubicin effects in vitro using AB vs. cell counting, which could be a consequence of potential doxorubicin-induced metabolic alterations. In contrast to in vivo results, 3D spheroids were sensitive to rapamycin and doxorubicin, and only doxycycline treatment could not significantly reduce the proliferation (ULA and HD cultures). The results obtained from 3D spheroids and 2D cultures show a higher similarity; however, these also differed from in vivo situations (Figure 2c). Comparing certain aspects of mTOR activity and other metabolic protein expression patterns by Wes^TM^ Simple in these models (2D cell culture, 3D spheroid and xenograft tissues), the detected expressions showed highly altered mTOR activity (Figure 2d). The highest mTOR activity was detected in 3D spheroids, and the C1–C2 complex activity distributions were characteristically different between the in vitro and in vivo systems. In the xenograft model, the cells grown in vivo showed higher mTORC1 activity-related S6K activation (p-S6) and a decrease in mTORC2 activity (p-Ser473-Akt). However, in the in vitro 2D and 3D cell culture systems, mTOR activity (p-mTOR) was correlated with a high level of mTORC2 complex activation (p-Ser473-Akt) without detectable mTORC1-related S6 kinase activity and remaining 4EBP1 phosphorylation independently of in vitro 2D or 3D structures. These alterations underline the consequences and role of the microenvironmental adaptation that occurred in ZR75.1 cells with balanced metabolic characteristics. Other studied metabolic protein expressions were also more similar in 3D spheroids and traditional 2D cell cultures and differed from those in tumours growing in vivo.

### 2.3. Growth, Morphological Characteristics and Drug Sensitivity of 3D Bioprinted ZR75.1 Tissue-Mimetic Scaffolds

**Figure 3:**
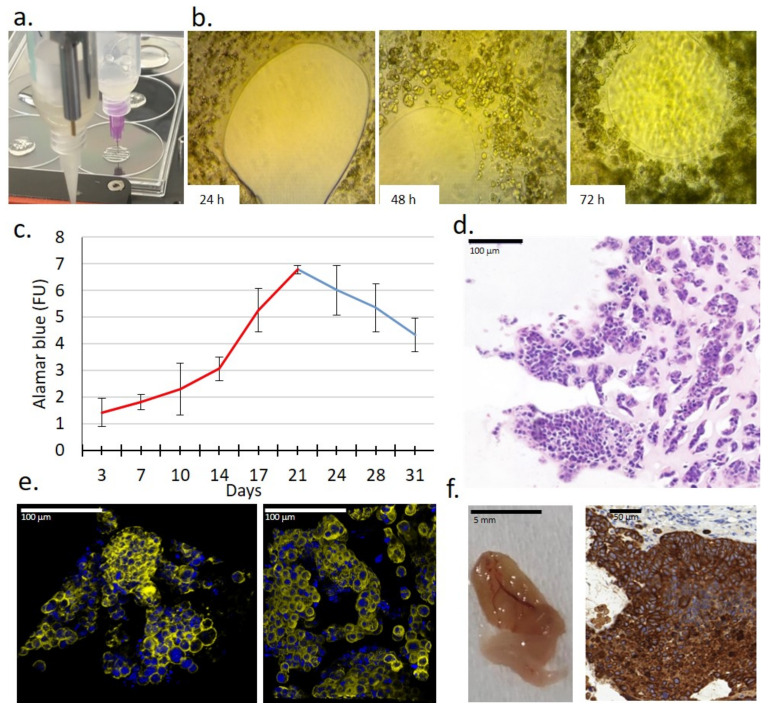
Characteristics of a new 3D bioprinted tissue-mimetic model of ZR75.1 human cancer cells. (**a**) Two dispenser units operated individually for constructing 3D bioprinted tissue-mimetic structures. (**b**) Cell growth of ZR75.1 cells was observed in 3D bioprinted tissue-mimetic scaffolds (TMSs) using an inverted microscope (Olympus CK-2) with photo documentation (magnification: 10×) after 24-, 48-, and 72-h culturing. (**c**) Registering the time-dependent in vitro growth of 3D bioprinted ZR75.1 TMSs by Alamar blue (AB) assay. In the first 3 weeks of maintaining TMSs, the cells were grown significantly, which was verified by their increasing metabolic activity. (**d**) Haematoxylin–eosin (H&E) staining of 3D bioprinted ZR75.1 TMS using paraffin-embedded sections. Scale bar is given in the figure. (**e**) Developed luminal structures in the 3D bioprinted ZR75.1 TMSs grown in vitro (phalloidin (yellow) and DAPI (blue) staining) detected by confocal microscopy (Leica Sp8 Lightning—LAS X Software) in control (left) and rapamycin + doxycycline-treated (right) TMSs. Scale bar is given in the figure. (**f**) Tumourigenicity of 3D bioprinted ZR75.1 TMSs was verified. A representative photo of vascularised 3D bioprinted TMS grown in vivo and its cytokeratin immunostaining positivity were detected. 3:3′-diaminobenzidine (DAB) (brown) was used as a chromogen.

In our recent study, we established a new 3D culturing method using 3D bioprinted ZR75.1 TMSs grown in vitro, and compared the detected sensitivity to other models (2D cell culture, 3D spheroids in ULA plates and in vivo xenografts) treated by rapamycin, doxycycline, doxorubicin and their combinations. For 3D bioprinting, alginate-based cell-free scaffolds and hydrogels containing ZR75.1 cells were printed as separate layers (Figure 3a,b). These gel compositions were selected based on a previous publication [42]. The time-dependent cellular growth of ZR75.1 cells was followed after 3, 7 or 10 days, or longer, of culturing (Figure 3c). TMSs were used freshly or fixed and several morphology studies were carried out. The 10/14-day-old TMSs showed morphological similarities to tissue sections based on haematoxylin–eosin (H&E) staining. Additionally, luminal structures formed in printed and cultured TMSs were detected with confocal microscopic observations (using phalloidin and DAPI staining) (Figure 3d,e, Video S1). The tumourigenecity of the printed TMSs was also tested. The xenotransplanted TMSs started to grow and their vascularisation was also found in SCID mice. The TMSs grown in vivo preserved their human BC origin, which was confirmed by IHC (e.g., human cytokeratin expression) after 1–2 months of growing (Figure 3f).

**Figure 4:**
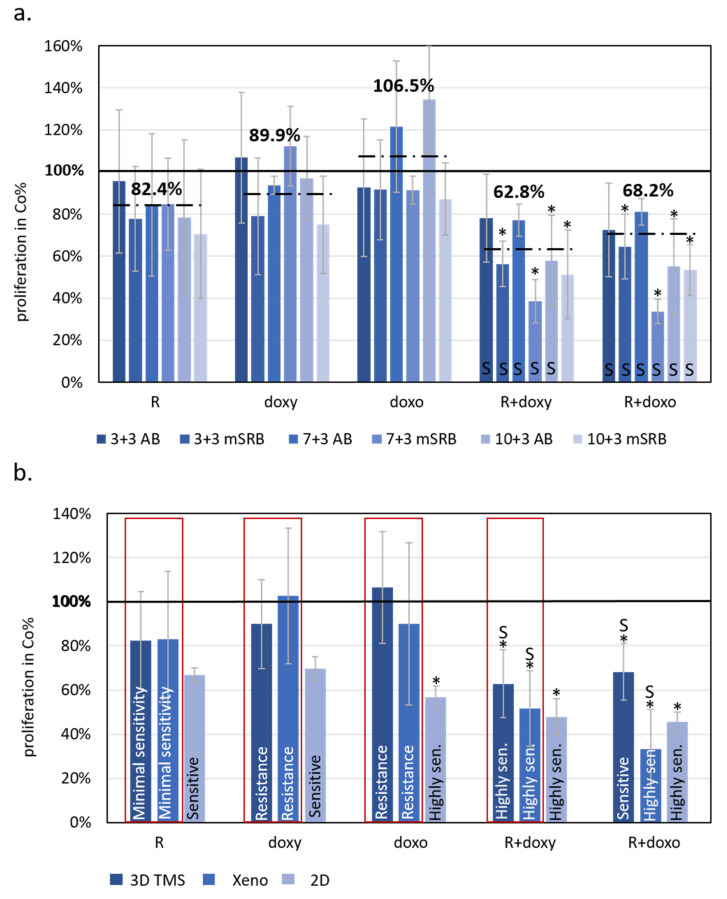
The effects of rapamycin, doxycycline and doxorubicin treatments showed similarities to 3D bioprinted TMSs grown in vitro and in vivo xenograft models of ZR75.1 breast cancer cells. (**a**) Alamar blue (AB) and modified sulforhodamine B (mSRB) assays were applied to follow the time-dependent sensitivity differences of ZR75.1 3D bioprinted tissue-mimetic scaffolds (TMSs) after rapamycin, doxycycline and doxorubicin mono- and combination treatments. The treatment period was 72 h after 3/7/10-day pre-culturing (3 + 3, 7 + 3 and 10 + 3, respectively). Dashed lines show average viability in control % calculated from the three different starting points and two different proliferation tests (six for each different drug treatment). (**b**) Compared results of the treatment responses in 2D, 3D bioprinted and xenografted ZR75.1 cells. Red frame shows the similarities of the sensitivity of the studied 3D bioprinted ZR75.1 TMSs (3D TMS) and xenografts (Xeno). The bars represent the average results from the performed experiments and analyses given in control % (thick black line—100%), and the sensitivity evaluation is also indicated in the chart. Sensitivity evaluation was given based on the altered proliferation: resistance ~90%; minimal sensitivity > 80%; sensitive 60–80%; highly sensitive < 60%. (Co—control; R—rapamycin/Rapamune, 50 ng/mL–3 mg/kg; doxy—doxycycline, 10 µM–5 mg/kg; doxo—doxorubicin, 50 ng/mL–2 mg/kg). Synergistic treatment interaction is labelled with S (based on combination index calculation). * *p* < 0.05.

After culturing the bioprinted TMSs for 3, 7, and 10 days in vitro, various analyses and 72 h drug tests were also applied. The detected sensitivity was analysed in comparison with the in vivo tumour growth of ZR75.1 xenografts and ULA-plate 3D spheroids using the same drug treatments. The proliferation/viability results in bioprinted TMSs using AB and SRB assays showed minimal rapamycin sensitivity, as well as doxycycline and doxorubicin resistance, as we detected in in vivo xenograft experiments. It was also confirmed in all of the studied model systems that the rapamycin combinations could significantly increase the sensitivity of both metabolic and chemotherapeutic drugs in ZR75.1 BC cells (Figure 4). Moreover, the synergistic effects of the treatment combinations were confirmed by the combination index calculations in the case of 3D TMS and xenograft models, respectively. Based on these comparisons, 3D bioprinted in vitro TMSs showed the highest similarity to in vivo conditions based on the tested drug sensitivity experiments.

**Figure 5:**
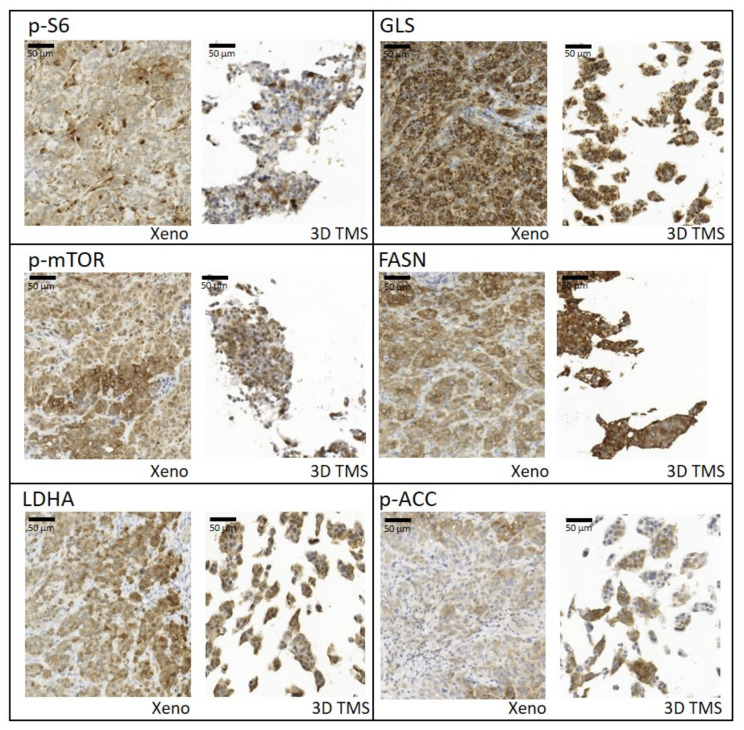
Tissue heterogeneity of metabolic enzyme expression and similarities between xenografts (Xeno) and 3D bioprinted tissue-mimetic scaffolds (3D TMS) of ZR75.1 cells. Immunostainings with anti-p-S6, -GLS, -p-mTOR, -FASN (fatty acid synthase), -LDHA (lactate dehydrogenase A), and -p-ACC (acetyl-CoA carboxylase) antibodies using 3,3′-diaminobenzidine (DAB) (brown) chromogen and haematoxylin counterstaining. Scale bar is given in the first figure.

Based on our in vitro studies, we detected a) that the characteristics of metabolic enzyme expressions of in vitro 2D and 3D spheroid cultures are closer to each other than to the xenograft model; and b) the highest similarity in terms of drug sensitivity was between TMSs and xenografts. Additionally, we focused on the in situ metabolic enzyme expression pattern, and we analysed how tissue heterogeneity was able to develop in the printed tissue-forming materials. IHC was performed on the formalin-fixed and paraffin-embedded 3D bioprinted TMSs and xenografts to study the tissue and tissue-mimetic heterogeneity of several previously studied metabolic enzymes. The expression patterns of several metabolic proteins and their heterogeneous staining in 3D bioprinted and xenografted tumours shared similarities in most of the applied stainings. Our pathologists scored the intensities of six stainings (p-S6, GLS, p-mTOR, FASN, LDHA, p-ACC), as H-scoring is usually performed in pathomorphology IHC studies. From these results, we calculated the Shannon Diversity Index (SDI) of stainings in both xenografts and TMSs. In human tissues, GLS expression was homogenous, and we could confirm this in our xenograft and 3D bioprinted TMSs, as well (the SDI was lower; 0.33 in both cases), without detectable heterogeneity. In contrast, the majority of metabolic enzymes showed intra-tumoural heterogeneous expression, as we expected (e.g., p-mTOR, FASN, p-ACC, p-S6), based on human IHC analyses. SDI indexes were almost similar and showed high intra-tumoural heterogeneous stainings of p-S6 (SDI = 1.03/1.17), p-mTOR (SDI = 1.16/1.22), FASN (SDI = 0.9/1.09), and p-ACC (SDI = 1.22/1.28) both in the xenograft and TMS slides, respectively. It was also found that LDHA regarding metabolic adaptation mechanisms showed higher heterogeneity (SDI = 1.09) in the 3D bioprinted TMS and xenograft experimental models than the representative diagnostic human specimens (Figure 5). These results confirm that the expression profile and the in vivo “tissue” heterogeneity of metabolic enzymes appear in bioprinted TMSs.

## 3. Discussion

The contribution of breast cancer to cancer-related mortality (6.9%) and its high inter- and intra-tumoural tissue heterogeneity are well-described in different studies [43,44]. Several new therapeutic options have been developed; however, the high number of relapsed and metastatic BC cases highlight that additional and better targeting methods are needed. Despite technological developments (using artificial intelligence and cutting-edge technologies), successful drug translations still need to be improved (less than 10% of drug candidates can enter clinical trials from animal experiments, and only 3% of these phase trials obtain approval) [44,45,46] in cancer drug development. Traditional 2D cell culturing models have made an enormous contribution to cancer research and therapeutic target development; however, tumour tissue heterogeneity cannot be well-studied in these models without a real 3D microenvironment.

There are several animal models (e.g., xenografts in SCID mice, and patient-derived tissue models), including genetically modified mice [47], where tumour development and progression are represented in 3D tissue-like structures, but such models lack the features of human cells (tumour and/or normal cells, model-type dependent) and/or the human microenvironment. Among other types of cancer cell lines, many established human BC cell lines are suitable for different in vitro experiments. Tumour cell lines—including the currently studied BC line—or primary isolated cells can be used in 3D cell culturing and 3D bioprinted models [5,48,49]. The continuously developing 3D cell culture technologies need detailed characterisation and optimisation as preclinical systems; higher reproducibility and standardisation are necessary for achieving more adequate experimental results and drug preselection.

The tumour microenvironment (e.g., the O_2_, nutrient concentration gradients, and hypoxia) has a special regulatory role in tumour growth and survival. The hypoxia responses can alter the metabolic activity of tissues, influence tumour tissue characteristics, decrease mitochondrial O_2_ consumption, increase Warburg glycolysis, and induce angiogenesis and the metastatic potential of the tumour population [50]. To model these characteristics in vitro, hypoxic chambers and/or several different microfluidic combined cell culturing systems can be applied. Using these, we can build hypoxic cores; however, mimicking the real tissue distribution of oxygen is almost impossible [51,52]. Using gel/scaffold base technologies, vessel-like pore networks can also be applied [52]. After building and treating these models, tissue-like structures can and must be analysed and studied by several morphology and molecular technologies. In these analyses, the cell recovery could face difficulties, and so this field needs development as well. There are several ideas, e.g., constructing 3D tumours by rolling a scaffold–tumour composite strip, and after the experiments unrolling the strip and rapidly disassembling it to perform snapshot analysis for, e.g., DNA/RNA, protein isolation or metabolic analysis [53]. To increase the cellular heterogeneity and complete these models, co-culture systems and multicellular organoid have started to be established with, e.g., fibroblasts, cancer-associated fibroblasts, adipocytes, and vessels. However, these models are extremely unstable and their long-term treatments face several difficulties [19,23,50,54]. There are some data about the treatment sensitivity differences of these models, which highlight that 3D structures and cellular complexity could influence the experimental results of in vitro therapeutic drug sensitivity tests [51,55]. Gene expression analyses data from these 2D vs. 3D spheroid cultures described some well-known and expected alterations in glycolysis, PI3K/Akt pathways and/or ECM protein and other metabolic events (e.g., mitophagy) and highlighted the importance of metabolic rewiring differences in the used in vitro models [51,55,56]. The cellular and ECM heterogeneity could have particular significance in immune-oncology drug tests and their in vitro experiments in future studies, as several papers suggested [52,53]. These findings all draw attention to the necessity of developing better, more in vivo-like model systems.

Despite available new models and multidisciplinary approaches, appropriate comprehensive studies which compare different models (2D, 3D spheroid, 3D bioprinted and in vivo) from the same human cell lines are still missing [57].

**Figure 6:**
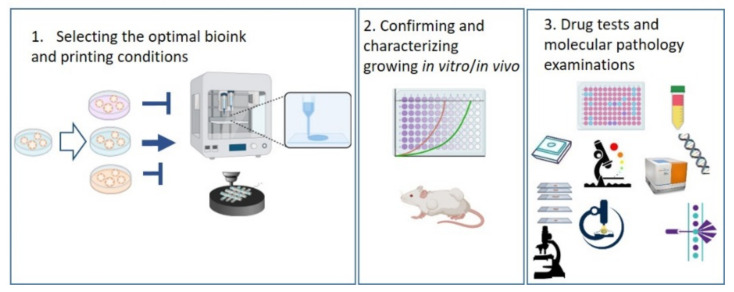
Sematic workflow of the established 3D bioprinted tissue-mimetic structures and their uses in different drug and molecular biology tests. Further details can be found in the text.

In our current investigations, we intended to create a comparative analysis including traditional 2D cultured, 3D cultured, 3D bioprinted TMSs and ZR75.1 human BC cells grown in vivo. We first compared the tissue formation morphology, different drug and drug combination sensitivities, protein expressions and in situ immunostaining patterns regarding tissue heterogeneity in all of our different non-multicellular model systems, including 3D bioprinted long-term cultured and treated models. According to our findings, 3D bioprinted TMS was the most relevant, in vivo-like system, among those studied using the same tumour cell line in vitro, which indicates that the established and described 3D bioprinting protocol and TMS are appropriate alternatives to replace and even improve traditional 2D and 3D cultures and reduce the number of animal experiments. In our 3D bioprinting workflow, we selected the optimal bioink composition and printing parameters for the used cell lines, following the morphology/growth/viability/proliferation of the cells in TMSs. There are many possibilities for registering the biological activity of the cells, including morphological observations, live/dead assays, protein or cell content and cellular metabolic activity analyses; we applied many of these in our experiments. The tumourigenicity of TMS was also confirmed by implanting the TMS structures into SCID mice to follow the in vivo growth. Our established model was also applicable for various drug tests, and molecular and pathomorphology-related research purposes (Figure 6).

Many 3D spheroid and biofabricated 3D models have been described with different origins, using BC cells. In most of these studies, the less aggressive MCF7 (Luminal A), or some triple-negative BC cell lines (MDA-MB-231 or MDA-MB-468) were used [58,59,60,61,62,63]. ZR75.1 cells (Luminal B type), which were the focus of our investigations, have the lumen-forming capability and an aggressive phenotype. We could confirm that the cell–cell connections, lumen formation and tissue-mimetic heterogeneity (studied protein expression pattern) also occurred under our long-term culturing conditions using 3D bioprinting technology. Various bioinks/hydrogels have been tested in different spheroid- and scaffold-printing studies [64,65]. Based on our pilot studies, alginate-based hydrogel was selected for human BC cells, similarly to some previously published pioneer works in 3D spheroid printing [58,59,61]. Alginate-based bioinks stabilized by CaCl_2_ are preferable for human cell printing avoiding mutagenic UV crosslinking (e.g., GELMA/edEMA) [66]. In the previous studies using BC cell lines and mainly 3D spheroid bioprinting, the authors described the growth characteristics, size and shape distribution [67] of the spheroids (live/dead cell ratio measured by different assays) and analysed some protein expressions (MMPs, EMT markers, stem cell markers, integrins, cell–cell connections, and matrix depositions) [58,68,69,70] or certain drug sensitivities (e.g., doxorubicin, paclitaxel, and 5-FU). In the case of doxorubicin treatment, maintained or decreased sensitivity were documented in 3D culture conditions [36,37,49,60,69,70,71,72]. These contradictions can be explained by the limitations of the used proliferation/viability assays, cell types and the differences between the used cell line and 3D culturing techniques in previous studies. Live/dead assays (calcein/propidium iodide) should be critically considered in these drug tests since the possible induced/altered expression of the MDR transporter (calcein is a substrate of MDRs) could influence the tests results in MDR-expressing cells [73]. Therefore, in our 3D TMSs, we applied both metabolic activity and total protein measurements to address this limitation (e.g., considering the potential metabolic adaptations—metabolic activity-based measurements in MTT; protein content of dead cells in SRB assays) and avoid misinterpretation using only one assay. Additionally, the novelty of our work is that we compared 2D spheroid, 3D spheroids, 3D bioprinted cultures and the xenografts grown in vivo in several aspects, including drug sensitivity, as well as the expression and distribution of certain metabolic proteins. Based on these comparisons, the 3D bioprinted TMS models have the highest similarity (better than 3D spheroids) to the in vivo situation. In addition to doxorubicin, two other compounds—the mTOR inhibitor rapamycin and doxycycline antibiotics with a mitochondrial inhibitory function—were tested in mono- and combined treatments. In the case of each monotherapy, the in vivo detected resistance was verified in 3D bioprinted TMSs in contrast to monolayer and 3D spheroid cultures. Higher sensitivity was detected in 2D cell cultures (all treatments were effective) and decreased but still-present sensitivities to rapamycin and doxorubicin were found in 3D spheroids in contrast with the in vivo and 3D bioprinted situations. Rapamycin and doxycycline have metabolic rewiring effects; therefore, their effects could be altered completely in 3D structures, where the metabolic activity is known to be variable [74,75]. Other results and studies call for using 3D culturing or in vivo systems to analyse the mTOR inhibitory or other regulatory effects on cellular metabolism [76,77]. In our presented quantitative comparative protein expression analyses, some similarities in cellular metabolic protein expressions in 2D cultures and 3D spheroids were underlined by Wes^TM^ Simple. Moreover, the developed tissue heterogeneity of the majority of the studied metabolic proteins was also confirmed in the xenografts and TMS samples grown in vivo. These findings are in harmony with the presented staining pattern heterogeneity of human BC specimens. In summary, the 3D bioprinting technology could help to establish new tissue-like in vitro cancer models, which are very close to the in vivo situation.

Summarising the detected metabolic differences of our models, the described important observations using traditionally (in 2D cell culture) metabolically balanced (with mainly OXPHOS characteristics) ZR75.1 cells were the following: (a) lowered mTOR activity shifted to the mTORC1 complex (regarding mTORC2 activity, the related p-Ser473-Akt level decreased, and the remaining mTOR activity was realized in mTORC1 activity) in 3D bioprinted TMS and xenograft cells, (b) the cells have individual in situ metabolic protein expression patterns (metabolic heterogeneity at the cellular level) in 3D bioprinted TMS and xenograft “tissues” vs. mainly monomorphic expression in 2D cultured cells, (c) metabolic inhibitors (rapamycin, doxycycline and their combinations) have similar effects in 3D bioprinted TMS and xenografts but showed higher sensitivity in the 2D and 3D spheroid cultures, and (d) increasing LDHB expression in TMSs and xenografts highlight the potential metabolic symbiosis in the tumour cell population, but this well-known in vivo mechanism was not registered in 2D and 3D spheroid cultures. Based on these findings, the detected metabolic differences could have a high impact on the therapeutic sensitivity of various model systems using the same parental cell lines. Some recent observations are in good correlation with these findings. For example, the reverse Warburg capacity of the cultured tumour cells, co-cultured tumour cells and fibroblasts were described in 3D co-cultures [78,79,80]. Regarding metabolic pathway analyses, other authors summarised the results of 2D and 3D cultured cell experiments in their papers; no drastically different situation was found in biofabricated “tissues” compared with those grown in vivo. In these papers, in correlation with our findings, altered metabolic processes (e.g., altered glycolytic, mTOR, and lipid metabolism activity) were highlighted, accompanied by lowered proliferation [81]. In harmony with our findings, they also suggest that 3D tissues could be better models to conduct in vivo functionality tests.

Therapy resistance, as well as tumour development and evolution, has very complex mechanisms. The presented novel 3D bioprinted models offer new possibilities for building real in vivo complexity in 3D structures. Patient/tumour-derived xenograft models and/or 3D bioprinting and in vitro short- or long-term culturing could be an effective platform for establishing precision cancer medicine. These studies have been started in some research groups in parallel with organoid cultures, who are developing the isolation of cells and the optimisation of bioinks, developing protocols to print and culture patient-derived 3D structures, and comparing in vitro and in vivo drug sensitivity with therapeutic treatment results [78,79,80]. These models also have advantages and disadvantages, and in addition, there are several summaries and reviews about these subjects [21]. Our further aim is to investigate 3D bioprinted breast, glioma and ovarian TMS specimens as novel approaches for investigating drug responses in our next studies, with clinical collaboration (after obtaining ethical approval).

To achieve tissue complexity, several cell types have to be integrated as organ- and function-dependent arrangements in future model development. New research directions have emerged toward tissue engineering, combining tumours and other cells (CAFs, vessels, adipocytes, etc.) with organ-on-chip and microfluidics technologies. The number of developments related to the organ-on-chip technique is increasing in many tumour types, including colon cancers, gliomas, breast cancers, osteosarcomas, lung cancers, etc. [65,68,69,70,71,72]. Modelling the complexity of tumour progression, especially the steps of metastasis formation, also requires microfluidics and several host tissues to be combined in novel in vitro test systems [82,83,84,85,86,87,88,89,90,91,92,93]. The continuously emerging data about such devices for BC and more complex BC models were summarised by *Moccia* and *Haase* [94,95]. Furthermore, we have to take into account that these bioengineered models and their increasing complexity are correlated inversely with adequate standardizations and validations, further elevating the costs. Finally, the constructed complexity could decrease the reproducibility and control of the focused biological mechanisms and study aims. Moreover, the use of 3D bioprinted tissue-mimetic structures (replacing monolayers and 2D co-cultures) has to be introduced and involved in chip models, especially in cancer research.

In conclusion, our presented investigations and the results from 3D bioprinted human breast cancer tissue-mimetic cultures provide additional steps in the direction of representing in vivo situations relating to cancer studies. According to the detected high similarity to the in vivo situation, the presented 3D bioprinted tumour tissue-mimetic structures and other combinations of cell printing and biofabrication could facilitate the further development of patient-derived drug tests, improve compound preselection in drug development studies and increase the success rate of clinical phase trials.

## 4. Materials and Methods

### 4.1. Studied Human Biopsies

Immunostaining was performed on resected tissue samples of breast cancer patients with different molecular subtypes (Luminal A—LumA, Luminal B—LumB, HER2+ and TN—triple negative; n = 10 for each). Patients were diagnosed at the Department of Pathology and Experimental Cancer Research, Semmelweis University, Budapest, Hungary between 2009 and 2017. The average age in the subtypes ranged between 59 and 68 at the time of diagnosis. After a minimum of a 5-year follow-up, 52.5% of the patients were alive, 2 patients had infiltrating lobular/ductal carcinoma and 2 had metastasis, according to the recently used standard protocol. All investigation protocols using human tissue samples were approved by the Institutional Ethical Review Board (SE KREB 216/2020.) and the Hungarian Scientific Council National Ethics Committee for Scientific Research (No. 7/2006).

### 4.2. Studied Human Cell Line and the Used Different in vitro Cell Culture Systems and 3D Bioprinting

ZR75.1 (ATCC—CRL1500), a human breast carcinoma cell line (Luminal B subtype), was used in our in vitro and in vivo experiments. Cells were maintained in RPMI 1640 media (Biosera—Nuaille, France) supplemented with 10% foetal bovine serum (FBS; Biosera), 2 mM L-glutamine (Biosera) and gentamycin (80 mg/2 mL; Sandoz, Basel, Switzerland) and stored at 37 °C in a humified atmosphere of 5% CO_2_. Media renewal or subculturing were carried out on every 2nd or 3rd day in traditional 2D cell culturing. Mycoplasma contamination was routinely checked using multiplex PCR [96] during our working period. To obtain a large number of cells for 3D bioprinting and xenograft experiments, the cells were maintained in T75 flasks (Sarstedt, Nümbrecht, Germany). For viability and proliferation response analyses (Alamar blue and sulforhodamine B assays; see below) 96-well plates were usually applied or the cell number was tested using the trypan blue dye exclusion method with a Bürker chamber. The initial seeding densities were the following: 7.5 × 10^4^ cells/15 mL/T75 flask and 2.5 × 10^3^ cells/100 µL/well of 96-well plates (Sarstedt). The next day after seeding or plating the cells, the media were renewed and the appropriate treatments were added for 72 h. Regarding the investigated treatment agents, the mTORC1 inhibitor rapamycin (R; 50 ng/mL; Focus Biomolecules, Plymouth Meeting, PA. USA), the antibiotic doxycycline hyclate (doxy; 10 µM; Merck-Sigma-Aldrich) and the chemotherapeutic agent doxorubicin (doxo; 50 ng/mL; TEVA, Debrecen, Hungary) were applied as monotreatments and combinations. The used concentrations were determined by our previous tests and literature data.

Combination index (CI) was calculated to predict the interactions of the used treatment agents. The following equitation was applied:

$$\mathrm{CI}=\frac{Ea+Eb}{Eab}$$

where *Ea* and *Eb* represent the detected effect of single monotreatments and *Eab* indicates the effect of the combined treatment. The assessed CI values can be used for classifying the interactions of treatment agents as follows: (a) synergistic effect (CI < 1); (b) additive effect (CI = 1); and (c) no additional effect (CI > 1) [40,97].

The formation of 3D spheroids was induced by two methods: hanging drop (HD) technique and ultra-low attachment (ULA) plates. To ensure that a single-cell suspension was used for spheroid formation, the cells were syringed with a 25 G needle before seeding. In the HD method, cells were seeded at a density of 1 × 10^4^/20 µL drops on the inner part of the inverted Petri dish (Sarstedt). To avoid droplet evaporation, the lower part of the Petri dish was filled with 15 mL phosphate-buffered saline (PBS) solution. The investigated treatment agents were added to the cells before droplet seeding. For establishing spheroids in 96-well ULA plates (Corning; Corning, NY, USA), 1 × 10^4^/100 µL cells were plated into each well. The following day, 100 µL of fresh media and the treatment agents were added to the wells. After a 72-h treatment, the cells growing in HDs or ULA plates were harvested, counted or processed for further research purposes.

The 3D bioprinted tissue-mimetic scaffolds (TMSs) were fabricated with the use of an extrusion-based bioprinter (Bioscaffolder 3.2, GeSiM, Radeberg, Germany). The layout of the structures was designed with GeSiM Robotics software. Cell-free and cell-containing hydrogel layers were printed in turns with the use of two dispenser units operated independently in different layers. The total layer number of the structures was 6 for in vitro proliferation tests and protein analysis. To prepare the printable bioink, cells at a density of 1 × 10^7^/mL were suspended in the mixture of hydrogels containing 3% alginate (Merck-Sigma-Aldrich, Darmstadt, Germany) and 1% gelatine (Merck-Sigma-Aldrich). A thicker scaffold hydrogel without cells was composed of 6% alginate and 11% methylcellulose (Merck-Sigma-Aldrich). The parameters of the bioprinting were as follows: scaffold radius: 2.5–5 mm; total height: 0.5–1 mm; interlayer angle: 90°; infill distance: 1.5 µm; layer connection: outline plus; printing speed: 10 mm/s; needle diameter and height: 110–110 µm (scaffold ink) or 50–50 µm (bioink with cells); and pressure: 425 kPa (scaffold bioink) or 20 kPa (bioink with cells). After printing, the structures were stabilised by crosslinking with 200 mM CaCl_2_ solution immediately to avoid dehydration, following incubation (2–5’ depending on the size of the structures). PBS was used to wash the samples, and finally the rafts were cultured for 3–31 days in media (refreshing was carried out every 3rd day). To perform proliferation assays, the scaffolds were transferred into 96-well plates, which were preloaded with 100 µL culturing media. The base and the treatment-induced alterations to cell growth/metabolic activity capacities were followed by Alamar blue assay (AB; Thermo Fisher Scientific, Waltham, MA, USA). In monolayer cultures, spheroids were generated in ULA plates and 3D bioprinted scaffolds, and 10% of the media were added from AB reagent to the wells at the 68th h of the treatment course. After a 4-h incubation period, the fluorescence was measured with a fluorimeter (Fluoroskan Ascent FL; Labsystems International; Ascent Software Version 2.6—Vantaa, Finland) at 570–590 nm. For registering the total protein content of the cells, the sulforhodamine B test (SRB, Merck-Sigma-Aldrich) was performed. Regarding monolayer cultures, 10% trichloroacetic acid (TCA; 50 µL/well, Merck-Sigma-Aldrich) was used for fixation. After a 1-h incubation at 4 °C, the fixative solution was discarded and the fixed cells were washed with distilled water to eliminate the traces of TCA. Then, 0.4 m/V% SRB dye was added (50 µL/well), followed by a 15-min incubation at room temperature. For washing out the unbound SRB, 1% acetic acid was used. The bound SRB was re-solubilised with the addition of 10 mM Tris base solution (150 µL/well, Merck-Sigma-Aldrich) to the wells. Finally, the absorbance was measured at 570 nm using the Multiskan MS microplate reader (Labsystems International; Transmit Software Version 4.5—Vantaa, Finland). In the case of 3D bioprinted scaffolds, the above-detailed SRB protocol was modified (mSRB) as follows: each washing step (distilled water and acetic acid) was carried out using a laboratory shaker to allow the washing solutions to pervade the entire structures. After washing, the scaffolds were left to dry out overnight, then the procedure was continued as described in the protocol used for monolayer cells with appropriate dilutions. Due to the characteristics of the 3D culturing methods, neither AB nor SRB test could be carried out in HD cultures. ULA plates are appropriate for performing AB assays, but SRB cannot be applied in liquid-phase cultures. Therefore, the number of cells was determined using a Bürker chamber after these experiments.

### 4.3. In Vivo Xenograft Model

To establish ZR75.1 xenografts, 2.5 × 10^6^ cells in 100 µL RPMI 1640 media (without FBS and antibiotic supplementations) or 3/7-day cultured TMSs were inoculated subcutaneously in the breast region of 8-week-old female SCID mice. Traditional engrafted tumours were used for treatments, which reached a palpable size after 3–5 weeks of injection. Then, the mice were divided into control and treated groups. The following treatment protocol proceeded in the following groups: group #1: control—saline/solvent solution intraperitoneal/intravenously/gavage; group #2: Rapamune (Pfizer—Budapest, Hungary; active ingredient: rapamycin) by gavage at 3 mg/kg body weight; group #3: doxycycline (Merck-Sigma-Aldrich) intravenously at 5 mg/kg body weight; group #4: rapamycin + doxycycline; and group #5: doxorubicin (TEVA—Debrecen, Hungary) intravenously at 2 mg/kg body weight. The treatments were administered three times a week. Tumour growth and the alteration of body weight were registered continuously. The following calculation method was used for assessing the tumour volumes:

$$\frac{\pi }{6}\times {\left(\frac{2\times shorter\;diameter+longer\;diameter}{3}\right)}^{3}$$

After a 21-day treatment period, the mice were sacrificed. The developed tumours were removed, then formalin-fixed and paraffin-embedded for performing further IHC analyses or were freshly lysed/frozen for further protein expression analyses. To determine the in vivo effects of treatment combinations, the CI method was used (the calculation formula is detailed in Section 4.2).

The in vivo experiments were conducted according to guidelines of the Institutional Animal Care Facility and approved by the Institutional Ethical Review Board (PE/EA/801-7/2020 approval date: 16 September 2020) with official permissions (PEI/001/1733-2/2015 approval date: 14 October 2015).

### 4.4. Protein Expression Studies—Immunohistochemistry and Wes^TM^ Simple Analyses

To perform immunohistochemistry (IHC) evaluations, formalin-fixed and paraffin-embedded human tissues, ZR75.1 xenograft tumours and 3D bioprinted ZR75.1 TMSs were also used. The deparaffinisation of sections was followed by antigen retrieval in a pressure cooker (usually in citrate buffer, pH = 6). After endogenous peroxidase blocking, the specimens were incubated with primary antibodies (the applied primary antibodies can be found in Table 2). Immunodetection was carried out with the Novolink Polymer Detection System (Novocastra), visualised by 3,3′-diaminobenzidine (DAB) staining (Aligent, Santa Clara, CA, USA) counterstained with haematoxylin. The human breast cancer origin of xenotransplanted 3D bioprinted TMSs was confirmed by using human cytokeratin staining with routine diagnostic immunohistochemistry antibody (cytokeratin—CKAE1 #M3515A; Dako, Aligent, Santa Clara, CA, USA; 1:150 dilution; after pH9 antigen retrieval) following Leica Bond Max DAB (Leica Biosystems, Buffalo Grove, IL, USA) chromogen detection. Two independent observers evaluated the immunoreactions and their heterogeneity using Pannoramic Viewer Software (3D Histech, Budapest, Hungary).

To check whether our obtained data about the IHC staining heterogeneity of breast cancer patients were correlated with others’ findings, the available collection of the Human Protein Atlas (Protein Atlas version 21.0, https://www.proteinatlas.org/ [98], access date: 11 May 2022) database was also evaluated. The data source allowed us to explore and compare the expression characteristics of various proteins, the protein expressions of which were available for use in our study (see Table 1).

Recently, in the IHC evaluations of some cancer-related studies, the Shannon Diversity Index (SDI) has been used to calculate the intratumoral heterogeneity of tumour tissue specimens. The calculation method is similar to that introduced and used in ecological-related studies to quantify the biodiversity (portion of individuals) of a species in a given dataset [99]. In a cancer-related context, the number/distribution of the tumour cell population can be estimated as follows [100]:

SDI = −∑ p_i_ ln(p_i_)

where p_i_ represents the percentage of each staining intensity in the tumour tissue.

In every case, the p_i_ was determined by two independent observers with the use of the histo-scores (H-scores) evaluation method [101]. To calculate an H-score, the staining intensity (0/+/++/+++) was multiplied by the percentage of positive cells for each staining intensity.

For further quantitative protein expression analyses, the cells, tissues and tissue TMSs were lysed with lysis buffer solution containing Tris (50 mM), glycerol (10%), NaCl (150 mM), Nonidet-P40 (1%), NaF (10 mM), phenylmethylsulfonyl fluoride (1 mM) and Na_3_VO_4_ (0.5 mM). The pH was adjusted to 7.5. The protein concentration of lysates was quantified by Bradford reagent (Bio-Rad, Hercules, CA, USA). A capillary-based Western system, Wes^TM^ Simple technique (ProteinSimple 004-600; Minneapolis, MN, USA), was used to determine the protein expression. The Anti-Rabbit Detection Kit (ProteinSimple DM-001) and Anti-Mouse Detection Kit (ProteinSimple DM-002) were applied depending on the used primary antibodies. In accordance with the molecular weight of the investigated antibodies, the detection was operated using a 12–230 kDa Separation Module (ProteinSimple SM-W004). Anti-β-actin was selected for initial loading control. The total list of the studied antibodies is included in Table 2. The steps of protein analysis were carried out based on the provided instructions of the manufacturer. The detailed description of the procedure can be found in our previous publications [102]. In short, cell lysates at a concentration range of 0.2–1 µg/µL and primary antibodies (1:50) were diluted. Next, Wes^TM^ Sample Buffer (ProteinSimple 042-195) and Fluorescent Master Mix (1:4, ProteinSimple PS-FL01-8) were added. After sample incubation (5 min, 95 °C), the Wes^TM^ capillary plate was loaded successively with primary and secondary antibodies as well as the chemiluminescent substrate mix (luminol/peroxide), respectively. The default parameter settings of each step were as follows: separation at 395 V for 30 min; blocking, 5 min; incubation with primary antibodies and secondary detection kit, 30–30 min; and luminol/peroxide detection, 15 min. Data evaluation was executed with Compass software version 6.1 (San Jose, CA, USA). The obtained electropherograms can be found as supplementary material (Figure S2).

### 4.5. Confocal Microscopy

Confocal microscopy was used to study the morphology of the developing 3D bioprinted and cultured TMSs. Fluorescent staining was performed using immunolabelling after 4% paraformaldehyde fixation, blocked with PBS containing 5% FBS, and supplemented with 0.3% Triton X-100. Following intracellular staining with fluorescent-labelled phalloidin-Atto550 (1:600; Merck-Sigma-Aldrich), Diamond antifade mountant with DAPI (1:600, #H3570, Invitrogen) was applied directly. The immunostaining was analysed using confocal microscopy (Leica Sp8 Lightning—LAS X Software Version 5.1.0.; Leica Microsystems).

### 4.6. Statistical Analyses

To calculate mean values and standard deviations (SD), the results of three independent experiments with three or more parallels (depending on the assays used) were evaluated. Statistical analyses of in vitro and in vivo experiments were performed using GraphPad Prism software (version 9.1.2; La Jolla, CA, USA). The post hoc multiple group comparisons were conducted using one-way ANOVA and two-way ANOVA (analysis of variance), followed by Tukey’s comparison tests. *p* ≤ 0.05 was considered statistically significant.

## Figures and Tables

**Table 1:** Tissue heterogeneity of several metabolic markers.

Evaluation of Staining Heterogeneity (Number of Heterogeneous Stainings/Total Studied Case Number %)							
	Performed IHC Analyses					Human Protein Atlas	
	LumA	LumB	HER2+	TNBC	Heterogeneity (%)	Heterogeneity (%)	Reference Antibody
**Rictor**	7/9	5/9	6/8	3/7	60%	42% (5/12)	HPA037802
**p-S6**	8/9	8/10	8/8	8/10	87%	n/a	n/a
**p-mTOR**	9/10	10/10	7/9	6/10	84%	n/a	n/a
**GLUT1**	4/10	6/10	9/9	7/10	67%	90% (10/11)	CAB002759
**LDHA**	2/10	4/10	3/9	2/10	28%	30% (4/12)	CAB015336
**HK2**	6/9	9/10	7/8	6/10	76%	30% (3/10)	HPA028587
**PFKP**	2/9	4/10	6/8	5/8	49%	46% (5/11)	HPA018257
**ASCT2**	8/10	10/10	8/8	9/10	92%	75% (9/12)	HPA035240
**GLS**	1/7	2/9	3/7	4/8	32%	18% (2/11)	HPA036223
**FASN**	4/10	6/10	5/9	4/9	45%	55% (6/11)	HPA006461
**CPT1A**	6/9	1/10	5/9	4/9	43%	30% (3/12)	HPA008835
**ACC**	4/8	6/10	6/6	3/8	59%	67% (8/12)	HPA063018
**ACSS2**	2/10	1/10	4/9	2/10	23%	33% (4/12)	HPA004141
**ATPb**	0/8	1/8	3/7	1/8	16%	50% (5/10)	HPA001528

Abbreviations: ACC—acetyl-CoA carboxylase; ASCT2—alanine, serine, cysteine-preferring transporter 2; ATPb—β-F1-ATP-ase; CPT1A—carnitine palmitoyltransferase 1A; FASN—fatty acid synthase; GLS—glutaminase; GLUT1—glucose transporter 1; HK2—hexokinase 2; LumA—Luminal A; LumB—Luminal B; LDHA—lactate dehydrogenase A; PFKP—phosphofructokinase; p-mTOR—phospho-Ser2448-mTOR; p-S6—phospho-Ser235/236-ribosomal S6; TNBC—triple-negative breast cancer. n/a—not available.

**Table 2:** Tissue heterogeneity of several metabolic markers. Primary antibody panel/list of the primary antibodies used for immunohistochemistry and Wes^TM^ analyses.

Primary Antibody	Abbreviation	Manufacturer	Catalogue Number	Dilutions		Target/Function/Marker
				Wes	IHC	
**Acetyl-CoA Carboxylase**	ACC	Cell Signaling	#3676	-	1:100	lipid metabolism; acetyl CoA carboxylation
**Acyl-CoA Synthetase Short-Chain Family Member 2**	ACSS2	Cell Signaling	#3658	-	1:200	acetate consumption
**Alanine, Serine, Cysteine-Preferring Transporter 2**	ASCT2	Bethyl	#A304-353A	-	1:250	glutamine transporter
**β-actin**		Merck-Sigma-Aldrich	#A2228	1:50	-	internal/loading control
**β-F1-ATPase**	ATPb	Abcam	#14730	-	1:100	mitochondrial oxidative phosphorylation
**Carnitine Palmitoyltransferase 1A**	CPT1A	Abcam	#128568	1:50	1:500	lipid metabolism; fatty acid beta-oxidation
**Cytochrome c oxidase subunit 4**	COX4	Cell Signaling	#4850	1:50	-	terminal oxidation reaction in the electron transport chain
**Fatty Acid Synthase**	FASN	Cell Signaling	#3180	1:50	1:100	lipid metabolism; fatty acid synthesis
**Glucose Transporter 1**	GLUT1	Abcam	#652	-	1:400	glucose transporter
**Glutaminase**	GLS	Abcam	#156876	-	1:200	glutaminolysis
**Hexokinase 2**	HK2	Cell Signaling	#2867	-	1:200	glycolysis
**Lactate Dehydrogenase A**	LDHA	Cell Signaling	#3582	-	1:400	glycolysis
**Lactate Dehydrogenase B**	LDHB	Abcam	#85319	1:50	-	glycolysis
**Phospho-The37/46-4EBP1**	p-4EBP1	Cell Signaling	#2855	1:50	-	mTOR complex activity
**Phosphofructokinase**	PFKP	Cell Signaling	#8164	-	1:100	glycolysis
**Phospho-Ser79-Acetyl-CoA Carboxylase**	p-ACC	Cell Signaling	#11818	-	1:100	lipid metabolism; acetyl CoA carboxylation
**Phospho-Ser235/236-Ribosomal S6**	p-S6	Cell Signalling	#4858	1:50	1:100	mTOR complex activity
**Phospho-Ser473-Akt**	p-Ser473-Akt	Cell Signaling	#4060	1:50	-	mTOR complex activity
**phospho-Ser2448-mTOR**	p-mTOR	Cell Signaling	#2976	1:50	1:100	mTOR complex activity
**Pyruvate Kinase Isoenzyme M2**	PKM2	Cell Signaling	#4053	1:50	-	glycolysis
**Rictor**		Bethyl	#A300-458A	-	1:1000	mTOR complex activity

## Data Availability

Publicly available datasets were analysed in this study. The data can be found here: Human Protein Atlas (Protein Atlas version 21.0); https://www.proteinatlas.org/.access date: 11 May 2022.

## Supplementary Materials

The following supporting information can be downloaded at: https://www.mdpi.com/article/10.3390/ijms23137444/s1.

## Abbreviations

ACC	acetyl-CoA carboxylase
ACSS2	acyl-CoA synthetase short-chain family member 2
ASCT2	alanine, serine, cysteine-preferring transporter 2
ATPb	β-F1-ATP-ase
BC	breast cancer
COX4	cytochrome c oxidase subunit 4
CPT1A	carnitine palmitoyltransferase 1A
DAB	3,3′-diaminobenzidine
Doxo	doxorubicin
Doxy	doxycycline hyclate
ECM	extracellular matrix
FASN	fatty acid synthase
FBS	foetal bovine serum
GLS	glutaminase
GLUT1	glucose transporter 1
HD	hanging drop
H&E	haematoxylin–eosin
HK2	hexokinase 2
IHC	immunohistochemistry
LDHA	lactate dehydrogenase A
LDHB	lactate dehydrogenase B
LumB	Luminal B subtype
MCT1	monocarboxylate transporter 1
mSRB	modified sulforhodamine B
p-4EBP1	phospho-The37/46-4EBP1
p-ACC	phospho- Ser79-acetyl-CoA carboxylase
PBS	phosphate-buffered saline
PDHB	pyruvate dehydrogenase E1 subunit beta
PFKP	phosphofructokinase
PKM2	pyruvate kinase isoenzyme M2
p-mTOR	phospho-Ser2448-mTOR
p-S6	phospho-Ser235/236-ribosomal S6
p-Ser473-Akt	phospho-Ser473-Akt
R	rapamycin/Rapamune
SDHA	succinate dehydrogenase complex flavoprotein subunit A
SRB	sulforhodamine B
TCA	trichloroacetic acid
TMS	tissue-mimetic scaffold
TN	triple-negative breast cancer
ULA	ultra-low attachment
Xeno	xenograft
