# Supplementary material for: Characterisation of 3D Bioprinted Human Breast Cancer Model for In Vitro Drug and Metabolic Targeting
Source: Int J Mol Sci. 2022 Jul 4;23(13):7444. doi: 10.3390/ijms23137444 (PMC9267600; doi:10.3390/ijms23137444)
Supplement: Supplementary file 1 [file ijms-23-07444-s001.zip › ijms-1771482-supplementary/Supplementary Figures.pdf]

International Journal of
Molecular Sciences

Article

Characterisation of 3D Bioprinted Human Breast Cancer Model for In Vitro Drug and Metabolic Targeting

Titanilla Dankó[1,†], Gábor Petővári[1,†], Regina Raffay[1], Dániel Sztankovics[1], Dorottya Moldvai[1], Enikő Vetlényi[1], Ildikó Krencz[1], András Rókusz[1], Krisztina Sipos[1], Tamás Visnovitz[2,3], Judit Pápay[1,‡], Anna Sebestyén[1,‡,*]

1 Department of Pathology and Experimental Cancer Research, Semmelweis University, Üllői út 26, H-1085 Budapest, Hungary; ; tita.danko@gmail.com (T.D.); gaborpetovari@gmail.com (G.P.); regiraffay@gmail.com (R.R.); sztankovics.daniel@gmail.com (D.S.); moldvai.dorottya@gmail.com (D.M.); eniko.vetlenyi@gmail.com (E.V.); krencz.ildiko@gmail.com (I.K.); rokusz.andras@med.semmelweis-univ.hu (A.R.); krisztina.sipos.13@gmail.com (K.S.); papay.judit@med.semmelweis-univ.hu (J.P.); hsebanna@gmail.com (A.S.)

2 Department of Genetics, Cell- and Immunobiology, Semmelweis University, Nagyvárad tér 4, H-1089 Budapest, Hungary; tamas.visnovitz@gmail.com (T.V.)

3 Department of Plant Physiology and Molecular Plant Biology, ELTE Eötvös Loránd University, Pázmány Péter sétány 1/c, H-1117, Budapest, Hungary; tamas.visnovitz@gmail.com (T.V.)

† contributed equally to the work as first authors

‡ contributed equally to the work as last authors

* Correspondence: sebestyen.anna@med.semmelweis-univ.hu and hsebanna@gmail.com (A.S.)

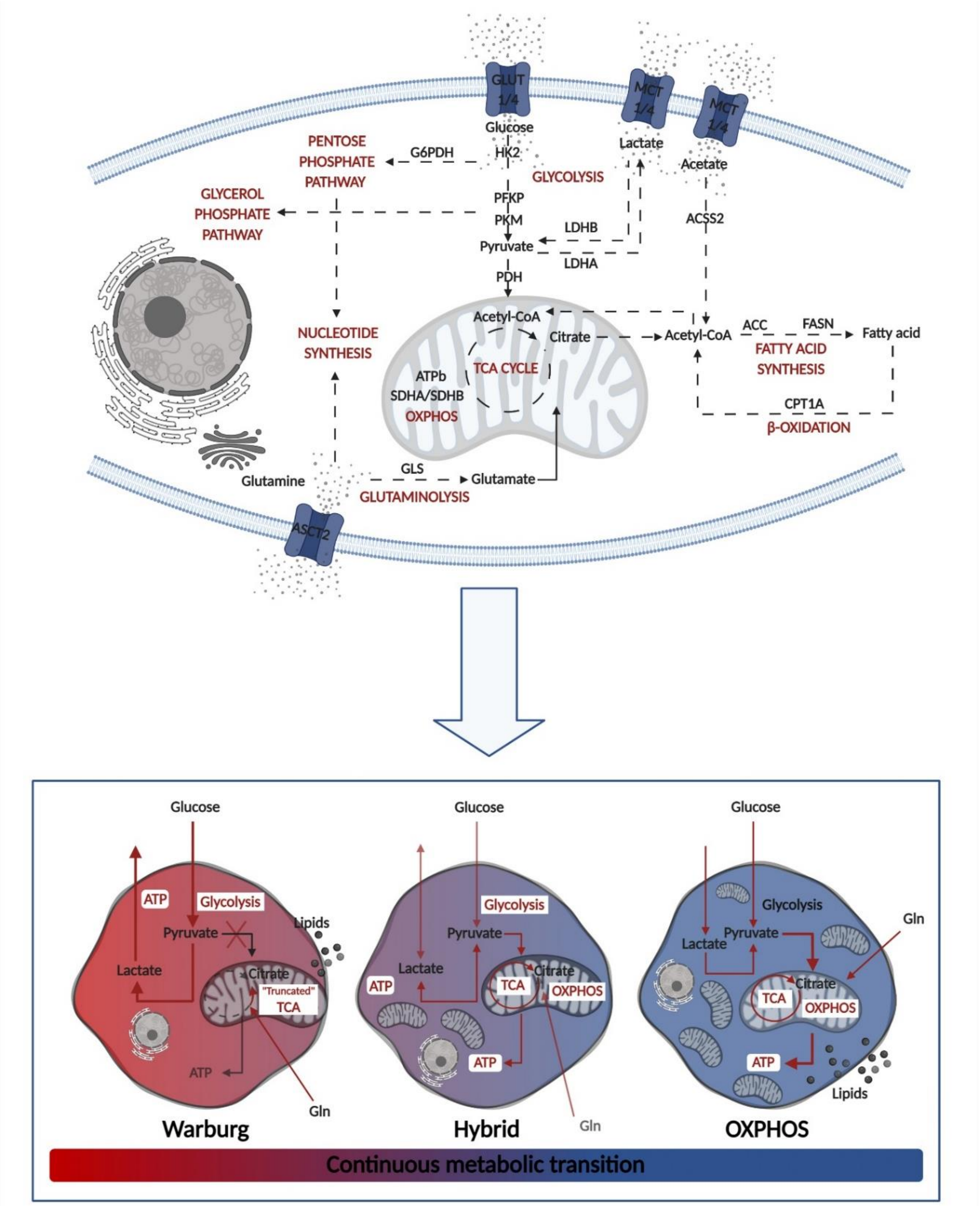

Figure S1. Schematic illustration of the studied metabolic enzymes and pathways. Alterations in cellular metabolism can initiate various metabolic phenotypes. A schematic simplified figure of the main metabolic pathways (the main pathways were indicated with red letters) and the studied elements of these were also highlighted. At the lower part of the figure, the simplified presentation of different metabolic phenotypes was shown. The metabolic rewiring contributes to continuous transition among Warburg, hybrid, and OXPHOS metabolic phenotypes in tumor tissues. The included figures were created with BioRender (https:// biorender.com) covered by the institutional license of the Department of Pathology and Experimental Cancer Research, Semmelweis University.

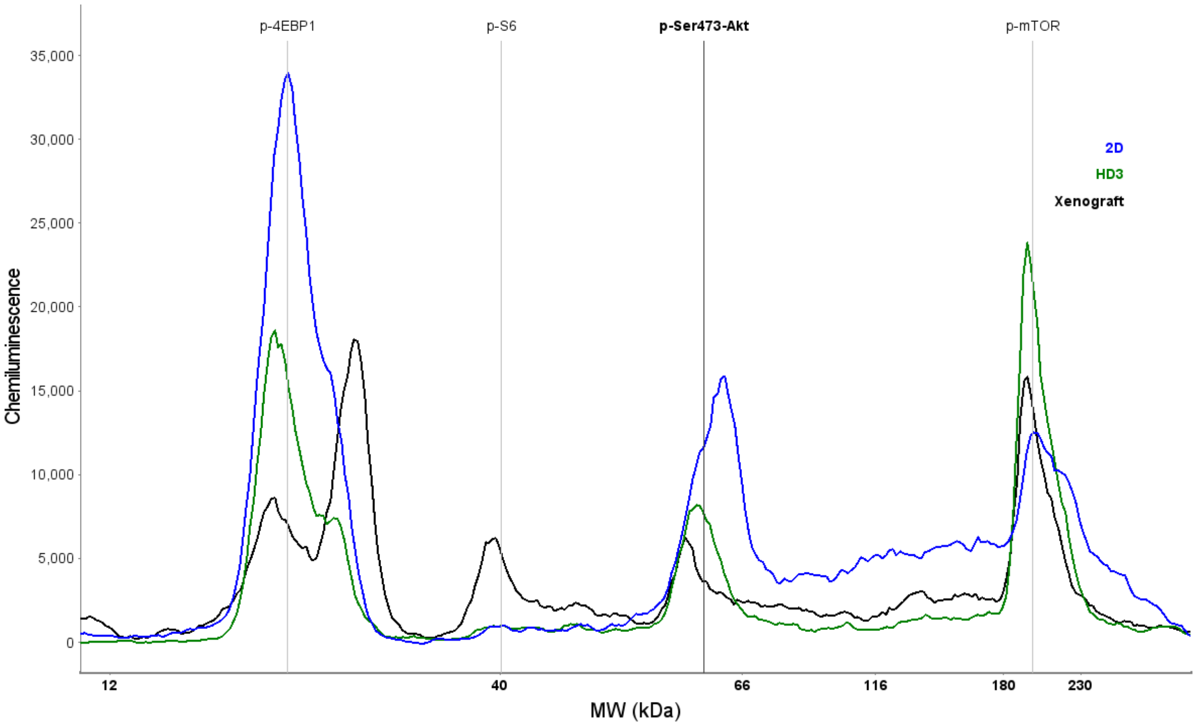
p-4EBP1
p-S6
p-Ser473-Akt
p-mTOR
35,000
30,000
25,000
20,000
15,000
10,000
5,000
0
Chemiluminescence
2D
HD3
Xenograft
12
40
66
116
180
230
MW (kDa)

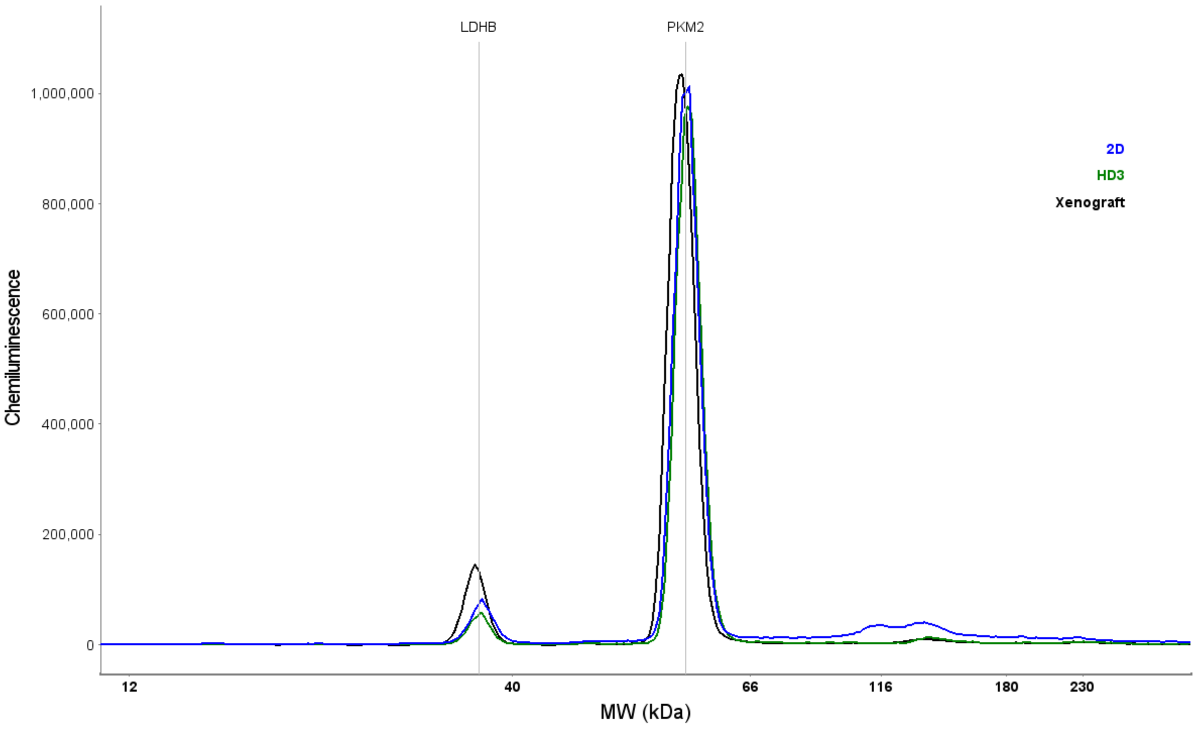
LDHB
PKM2
1,000,000
800,000
600,000
400,000
200,000
0
Chemiluminescence
2D
HD3
Xenograft
12
40
66
116
180
230
MW (kDa)

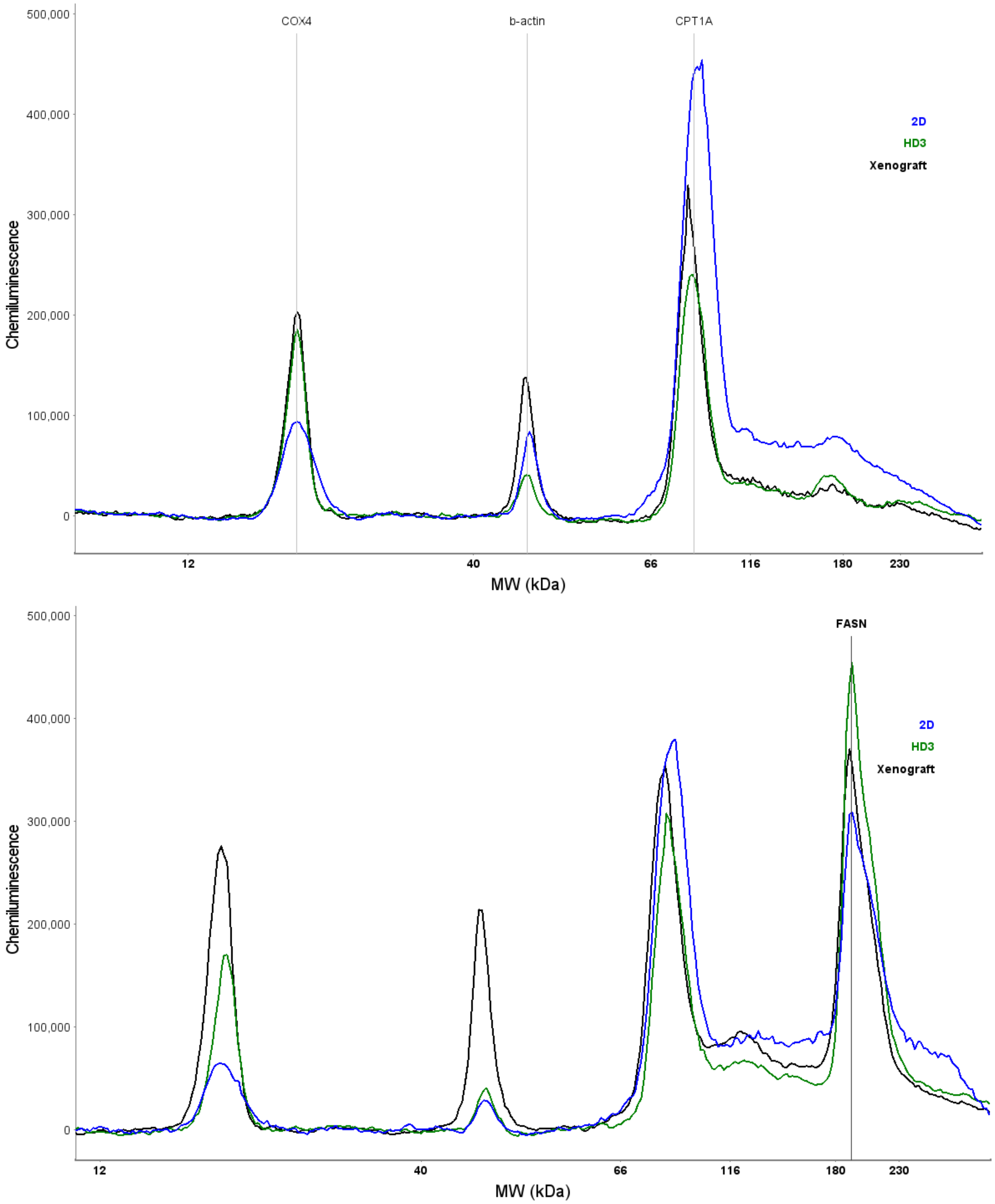

Figure S2. Additional information to WES Simple analyses.
